# *Escherichia coli* Heat-Labile Enterotoxin B Subunit Combined with Ginsenoside Rg1 as an Intranasal Adjuvant Triggers Type I Interferon Signaling Pathway and Enhances Adaptive Immune Responses to an Inactivated PRRSV Vaccine in ICR Mice

**DOI:** 10.3390/vaccines9030266

**Published:** 2021-03-16

**Authors:** Fei Su, Yige Wu, Junxing Li, Yee Huang, Bin Yu, Lihua Xu, Yin Xue, Chenwen Xiao, Xiufang Yuan

**Affiliations:** 1 Institute of Animal Husbandry and Veterinary Science, Zhejiang Academy of Agricultural Sciences, Hangzhou 310002, China; sufei@zaas.ac.cn (F.S.); d_count@126.com (Y.W.); lijunx@zaas.ac.cn (J.L.); huangye@zaas.ac.cn (Y.H.); yub@zaas.ac.cn (B.Y.); xulihua@zaas.ac.cn (L.X.); xiaochenwen@zaas.ac.cn (C.X.); 2 Zhejiang Center of Animal Disease Control, Hangzhou 310020, China; schroyen@163.com

**Keywords:** PRRSV, intranasal adjuvant, LTB, Rg1, type I interferon

## Abstract

Porcine reproductive and respiratory syndrome virus (PRRSV) is a major pathogen that has threatened the global swine industry for almost 30 years. Because current vaccines do not provide complete protection, exploration of new preventive strategies is urgently needed. Here, we combined a heat-labile enterotoxin B subunit of *Escherichia coli* (LTB) and ginsenoside Rg1 to form an intranasal adjuvant and evaluated its enhancement of immune responses in mice when added to an inactivated-PRRSV vaccine. The combination adjuvant synergistically elicited higher neutralizing and non-neutralizing (immunoglobulin G and A) antibody responses in the circulatory system and respiratory tract, and enhanced T and B lymphocyte proliferation, CD4^+^ T-cell priming, and cytotoxic CD4^+^ T cell activities in mononuclear cells from spleen and lung tissues when compared to the PRRSV vaccine alone, and it resulted in balanced Th1/Th2/Th17 responses. More importantly, we observed that the combination adjuvant also up-regulated type I interferon signaling, which may contribute to improvement in adaptive immune responses. These results highlight the potential value of a combined adjuvant approach for improving the efficacy of vaccination against PRRSV. Further study is required to evaluate the efficacy of this combined adjuvant in swine.

## 1. Introduction

Porcine reproductive and respiratory syndrome (PRRS) is one of the most severe swine diseases, characterized by reproductive failure in sows and respiratory disease in pigs of all ages [1,2]. The pathogenic agent is porcine reproductive and respiratory syndrome virus (PRRSV), which is an enveloped, positive-strand RNA virus of the family *Arteriviridae* [3]. PRRSV infection is highly restricted to cells in the monocyte/macrophage lineage such as porcine alveolar macrophages (PAMs), which are the primary targets of PRRSV in vivo [4]. The typical effects of PRRSV infection on the host immune system include persistent viremia, inhibition of innate cytokines (interferon (IFN)-α/β, tumor necrosis factor (TNF)-α, etc.), dysregulation of natural killer (NK) cell function, delayed appearance of neutralizing antibodies, and induction of regulatory T cells (Tregs) [4]. To prevent PRRSV infection, both live attenuated and inactivated PRRSV vaccines have been used for more than two decades; however, these vaccines have been unsuccessful in controlling PRRS. Live attenuated PRRSV vaccine confers protection only against homologous, but not heterologous viruses [5,6]. In addition, the risk of virulence reversion and shedding by attenuated strains limits its use [7]. In contrast, the PRRSV-inactivated vaccine has a good safety profile, but poor immunogenicity [8]. Therefore, more effective vaccination strategies for PRRSV prevention are urgently needed. One alternative solution is to combine adjuvant with vaccine formulations to improve the efficacy of vaccination [8]. 

Because the respiratory mucosal surface is the primary site for PRRSV transmission and infection, direct intranasal (IN) vaccination could be an effective strategy for protection against PRRSV by induction of systemic and mucosal immune responses [9]. The B subunit of *Escherichia coli* heat-labile enterotoxin (LTB) is known to be a non-toxic mucosal adjuvant for a range of vaccines [10,11,12], although the mechanism of adjuvanticity is not clear yet. It has been reported that IN immunization with the enterovirus 71 VP1 subunit (EVP1) plus LTB as adjuvant can significantly improve EVP1-specific systemic and mucosal antibodies in mice [13]. In addition, IN vaccination with the recombinant chimeric protein containing *Mycoplasma hyopneumoniae* three antigens (R1, P42, and NrdF) fused to LTB subunit strongly enhanced specific immune responses in mice and pigs [14]. Moreover, co-administration of recombinant fowl cholera outer membrane protein H (rOmpH) with LTB via IN route conferred 70% protection against challenge in chickens, compared with 0% protection in the rOmpH-only group [15]. These results indicate that LTB is a potent nasal adjuvant when co-administrated or fused with various antigens. GM1 ganglioside, a glycosphingolipid found ubiquitously on eukaryotic cell surfaces, is the major receptor for LTB [16]. Previous studies have suggested that GM1-binding activity is a necessary but not the only mechanism for LTB adjuvanticity [17,18]. Our previous work has demonstrated that LTB serves as a nasal adjuvant of an inactivated PRRSV vaccine by enhancing PRRSV-specific immune responses to Th1 type (T helper type 1) cells in mice [19]. Nevertheless, a Th1/Th2 balanced immunity is more desirable to provide potent and broad protection against pathogen invasion. Ginseng saponins, or ginsenosides, are considered one of the biologically active ingredients in ginseng extracts. More than 40 ginsenosides have been identified to date [20]. Our previous studies have demonstrated that ginsenoside Rg1 has adjuvant properties when administered parenterally and that its mechanisms are closely related to that of the Toll-like receptor 4 (TLR4) signaling pathway: adjuvant efficacy of Rg1 is absent in *Tlr4* mutant mice and Rg1-induced nuclear factor-kappa B (NF-κB) activity in macrophages is suppressed by blocking TLR4 [21,22]. However, the mucosal adjuvant properties of Rg1 remain unclear. 

In the present study, LTB and ginsenoside Rg1 (LTB-Rg1) were used together as a combination adjuvant and intranasally administered to mice with an inactivated PRRSV vaccine. Our findings demonstrate that LTB-Rg1 synergistically promotes PRRSV-specific systemic and respiratory immunoglobulin (Ig) G and IgA antibody responses, neutralizing antibody titers, lymphocyte proliferation, cytokine expression, and T cell activities. In addition, we found that LTB-Rg1 triggers type I interferon signaling activation, especially increases the expression of IFN-α protein, which is critical for the antiviral responses.

## 2. Materials and Methods

### 2.1. Adjuvants

Recombinant LTB was produced by the GS115 strain of *Pichia pastoris* as previously described [19]. Ginsenosides Rg1 extracted from the root of *Panax ginseng* was purchased from Biopurify Co. Ltd. (Chengdu, China). All solutions were passed through a gel endotoxin-removing column (Pierce, Shanghai, China), and the endotoxin level of solutions was below 0.5 EU/mL.

### 2.2. Animals

Female ICR (Institute of Cancer Research) mice aged 6–8 weeks (18–20 g) were obtained from the Shanghai Laboratory Animal Center. Mice were housed in polypropylene cages with sawdust bedding in a hygienically controlled environment with stable temperature (24 ± 1 °C). Feed and water were supplied ad libitum. All protocols related to animal care and use were approved by the Zhejiang Academy of Agricultural Sciences Institutional Animal Care Committee in accordance with the recommendations of the National Institutes of Health Guide for the Care and Use of Laboratory Animals (Ethics protocol No. 2020001)

### 2.3. Cells and Viruses

MARC-145 cells were incubated in Dulbecco’s modified Eagle’s medium (DMEM) containing 10% (*v*/*v*) fetal bovine serum (FBS) at 37 °C in 5% CO_2_. PRRSV strain JXA-1 was propagated and titrated in MARC-145 cells with 1 × 10^6^ TCID_50_ (tissue culture infectious dose 50)/mL. The virus was then inactivated using 0.05% β-propiolactone (Solarbio Life Sciences, Beijing, China) at 4 °C for 12 h, and another 2 h at 37 °C. Inactivation was confirmed by blindly passaging the treated virus three times in MARC-145 cells.

### 2.4. Experimental Design

**Figure 1:**
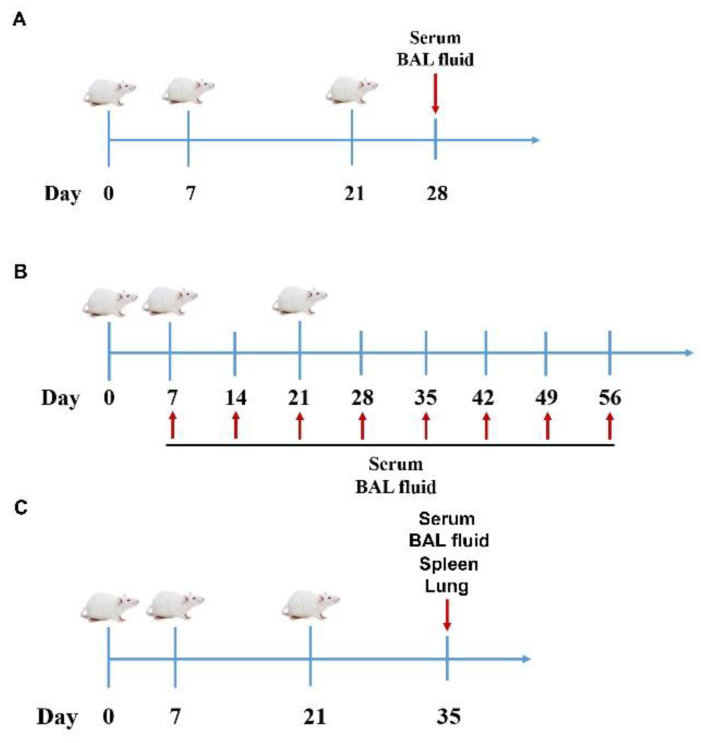
Immunization schemes. (**A**) Vaccination schedule of experiment A. Mice (*n* = 6/group) were immunized intranasally on days 0, 7, and 21. On day 28, serum and bronchoalveolar lavage (BAL) fluid were collected for antibody assays. (**B**) Vaccination schedule of experiment B. Mice (*n* = 48/group) were immunized intranasally on days 0, 7, and 21. On days 7, 14, 21, 28, 35, 42, 49, and 56, 6 mice were taken out from each group and euthanized. Serum and BAL fluid were collected to detect antibody responses. (**C**) Vaccination schedule of experiment C and D. Mice (*n* = 18/group in experiment C, *n* = 12/group in experiment D) were immunized intranasally on days 0, 7, and 21. On day 35, serum and BAL were collected for determination of IgG isotypes and neutralizing antibody titers; spleens and lungs were harvested for detecting IgA-secreting cells, lymphocyte proliferation, cytokine production, T cell differentiation, and type I interferon signaling activities.

In experiment A, mice (*n* = 6/group) were anesthetized with isofluorane and immunized intranasally (50 µL volume) with 1 × 10^5^ TCID_50_ (tissue culture infectious dose 50) inactivated PRRSV vaccine admixed with either saline, LTB (10, 20, 30 µg), Rg1 (10, 30, 50 µg), or LTB (10, 20, 30 µg) in combination with Rg1 (10, 30, 50 µg) on days 0, 7, and 21 (Figure 1A). Non-immunized mice (*n* = 6) served as negative controls (NC). On day 28, serum samples were collected for IgG antibody assays. Lungs were lavaged 5 times with 1 mL phosphate buffered saline (PBS) containing 3 mM EDTA to obtain bronchoalveolar lavage (BAL) fluid for IgA antibody assays. 

In experiment B, mice (*n* = 48/group) were anesthetized with isofluorane and immunized intranasally with 1 × 10^5^ TCID_50_ inactivated PRRSV vaccine admixed with either saline, LTB (20 µg), Rg1 (30 µg), or LTB (20 µg) plus Rg1 (30 µg) on days 0, 7, and 21 (Figure 1B). Non-immunized mice (*n* = 48) served as NC. On days 7, 14, 21, 28, 35, 42, 49, and 56, 6 mice were taken out from each group and euthanized. Sera and BAL fluid were collected to detect IgG and IgA responses. 

In experiment C, mice (*n* = 18/group) were grouped and vaccinated in the same way as experiment B (Figure 1C). All of the animals were euthanized on day 35. Sera and BAL fluid were collected to detect IgG isotype responses and neutralizing antibody titers. Six mice were taken out from each group, and the lungs were harvested and immediately fixed in 10% (*v*/*v*) neutral buffered formalin for immunohistochemical examination. Another 6 mice were taken out from each group, the spleens and lungs were harvested for determination of lymphocyte proliferation and quantification of interferon regulatory factor (IRF)3 mRNA expression. The remaining 6 mice in each group were dissected, and the spleens and lungs were collected for determination of IFN-α production and IRF3 phosphorylation.

In experiment D, mice (*n* = 12/group) received the same vaccination program as in Experiment B (Figure 1C), using 1 × 10^5^ TCID_50_ inactivated PRRSV vaccine admixed with either saline or LTB (20 µg) plus Rg1 (30 µg). Non-immunized mice (*n* = 12) served as NC. Spleens and lungs from 6 mice of each group were collected for either cytokine quantification or flow cytometry analysis.

### 2.5. Determination of PRRSV-Specific IgA, IgG, and Isotype Responses

PRRSV-specific IgA, IgG, IgG1, and IgG2a antibodies were measured by indirect enzyme-linked immunosorbent assays (ELISA) [19]. Briefly, polyvinyl 96-well microplates pre-coated with PRRSV antigen were obtained from IDEXX PRRS X3 ELISA kits (IDEXX, Westbrook, USA). After adding either 100 µL of diluted serum samples (1:100) or BAL fluid (1:10), the plates were incubated at 37 °C for 1h. After washing, 100 µL of horseradish peroxidase (HRP)-conjugated goat anti-mouse IgA (1:2000), IgG (1:5000), IgG1 (1:2000), or IgG2a (1:2000) was added and the plates were incubated at room temperature for 1 h. After another round of washing, 100 µL of 3, 3′,5,5′- tetramethyl benzidine substrate solution (IDEXX, Westbrook, USA) was added to each well, and the reaction was stopped using stopping solution after 15 min. The optical density (OD) of the plates was read at 450 nm by an ELISA plate reader (Thermo Multiscan MK3, Waltham, MA, USA). 

### 2.6. Immunohistochemical Staining for IgA-Secreting Cells

IgA^+^ cells were identified with the immunohistochemical staining method as described previously [23]. Briefly, fixed lung tissues were first embedded in paraffin, sectioned at 6 µm thickness, and mounted onto poly-lysine-coated glass slides. The slides were then washed with 0.01 M PBS (pH 7.4). Endogenous peroxidase activity was blocked by incubation with 3% H_2_O_2_ in methanol for 20 min. After rinsing three times with 0.01 M PBS (pH 7.4), the slides were then incubated sequentially with goat anti-mouse IgA antibody (1:400) and HRP-conjugated rabbit anti-goat IgG (1:250). 3,3′-diaminobenzidine (DAB) detection was performed according to the manufacturer’s instructions. After immunohistochemical staining, the slides were counterstained with hematoxylin, dehydrated with alcohol, cleared with xylene, and sealed with neutral gum. Images were captured with a Nikon DS-U3 camera interface and measured using image analysis software Image-Pro Plus 6.0. Three fields of each slide were randomly selected at ×200 magnification and the integrated optical density (IOD) was analyzed. The average value was calculated as the IOD value of the lung specimen.

### 2.7. Analysis of Neutralizing Antibodies

Antibody analysis was performed as previously described [24]. Briefly, all samples were heat-inactivated at 56 °C for 30 min and 1:2 serially diluted. The serial dilutions of samples were mixed with equal volumes of 100 TCID_50_ PRRSV JAX-1 strain and incubated at 37 °C for 1 h. The mixtures were then transferred to MARC-145 monolayers on 96-well plates. The plates were incubated for 5 days at 37 °C in a 5% CO_2_ atmosphere and the cytopathic effect (CPE) was recorded. Neutralizing antibody titers were determined by the Reed–Muench method.

### 2.8. Test of Lymphocyte Proliferation

Spleen and lung leukocytes were prepared as previously described [21,25]. Briefly, spleens were homogenized and passed through a steel mesh to obtain cell suspension. After centrifugation at 380 *g* for 10 min, the cell pellets were washed in Hank’s balanced salt solution and resuspended in complete RPMI 1640 medium (RPMI, 10% FBS, 100 IU/mL penicillin, and 100 µg/mL streptomycin). Lung tissues were minced into small pieces and incubated in digestion buffer containing 2 mg/mL collagenase type XI and 100 μg/mL DNase (Sigma-Aldrich, Shanghai, China) for 60 min at 37 °C. The cell suspension was gently mixed with 35% Percoll and centrifuged at 380 *g* for 20 min. The pellets were collected and re-suspended in complete RPMI 1640 medium. The erythrocytes were lysed with ammonium chloride-potassium (ACK) lysis buffer (Solarbio Life Sciences, Beijing, China) and re-suspended in complete RPMI 1640 medium. Mononuclear cells were seeded onto 96-well plates at 5 × 10^6^ cells/mL and stimulated with concanavalin (ConA, 5 µg/mL), liposaccharide (LPS, 8 µg/mL), purified PRRSV antigen (10 µg/mL), or medium alone. After incubation for 48 h, 10 µL of CCK-8 (DojinDo laboratories, Shanghai, China) was added and plates were sequentially incubated for 4h. Absorbance at 450 nm was evaluated with a microplate reader. The stimulation index (SI) was calculated based on the formula: OD value of mitogen cultures/OD value of non-stimulated cultures.

### 2.9. Cytokine Assay

Mononuclear cells from spleen and lung were re-stimulated with purified PRRSV antigen (10 µg/mL) for 72 h at 37 °C in a 5% CO_2_ atmosphere. The concentration of IFN-*γ*, IL-2, IL-5, IL-10, IL-6, IL-17A, and IFN-α in supernatants was determined using commercial ELISA kits (Neobioscience, Shanghai, China) according to the manufacturer’s instructions. 

### 2.10. Flow Cytometry Analysis

Mononuclear cells (5 × 10^6^ cells/mL) from spleen and lung were washed and stained with phenotyping antibodies for 30 min in the dark. The following antibodies were purchased from eBioscience and used for flow cytometry assay: FITC conjugated-CD3e (Clone 145-2C11), APC conjugated-CD4 (GK1.5), PerCP-Cy5.5 conjugated-CD8a (Clone 53-6.7), PE-conjugated CD107a (LAMP-1), and APC conjugated-IL-10. Mouse T-helper (Th) 1/Th2 and Th17 staining kits were purchased from MultiSciences (Hangzhou, China). FlowJo V10 software was used to analyze the data.

### 2.11. Quantitative Real-Time PCR (qPCR) 

Splenocytes and lung cells from immunized mice were seeded onto 24-well plates at 5 × 10^6^ cells/mL and re-stimulated with purified PRRSV antigen (10 µg/mL) in vitro. After incubation for 24 h, RNA was extracted using a Culture Cell Total RNA Extraction Kit (Simgen, Hangzhou, China). qPCR analysis was performed using TransScript^®^ II Green One-Step qRT-PCR SuperMix (Transgen, Shanghai, China) on ABI7500 real-time PCR system (Applied Biosystems, Shanghai, China). The following primers were used: IRF3 forward 5′-TCATCGAAGATCTGATTGCCTTC-3′ and reverse 5′-GGGACAACCTTGACCATCACC-3′; β-actin forward 5′-CAAGGACCTCTACGCCAACAC-3′ and reverse 5′-TGGAGGCGCGATGATCTT-3′. IRF3 expression was normalized to β-actin and presented as fold induction relative to the control using 2^-ΔΔCT^ method [26,27].

### 2.12. Western Blot Assay

Splenocytes and lung cells (5 × 10^6^ cells/mL) from immunized mice were re-stimulated with purified PRRSV antigen (10 µg/mL) in vitro and lysed using radio-immunoprecipitation assay (RIPA) lysis buffer supplemented with proteinase cocktail and phosphatase inhibitor (Beyotime, Shanghai, China). Protein concentration in each sample was quantified with a BCA reagent (Sangon Biotech, Shanghai, China). Equivalent protein concentrations from each extract were fractionated by SDS-PAGE and transferred onto an Immobilon-p transfer membrane (Millipore Corporation, USA). After blocking with 5% skimmed milk in TBST (TBS containing 0.1% Tween 20), membranes were incubated with primary antibodies (Cell Signaling Technology, Shanghai, China) at 4 °C overnight. The membranes were then incubated with the appropriate secondary antibodies (Abcam, Hangzhou, China) for 1 h at room temperature. The immunoblot was developed with a Pierce^TM^ ECL Western blotting substrate (Thermo Fisher, Shanghai, China) according to the manufacturer’s instructions, and the images were captured with a Chemi-Scope imaging system (Bio-Rad, Shanghai, China). 

### 2.13. Statistical Analysis

All statistical analyses were performed with GraphPad Prism 7.0 software (San Diego, CA, USA) by one-way ANOVA with Tukey’s multiple comparisons. Data were expressed as mean ± standard deviation (SD). A *p*-value < 0.05 was considered as a statistically significant difference. 

## 3. Results

### 3.1. LTB and Rg1 Synergistically Enhance PRRSV-Specific Antibody Responses

**Figure 2:**
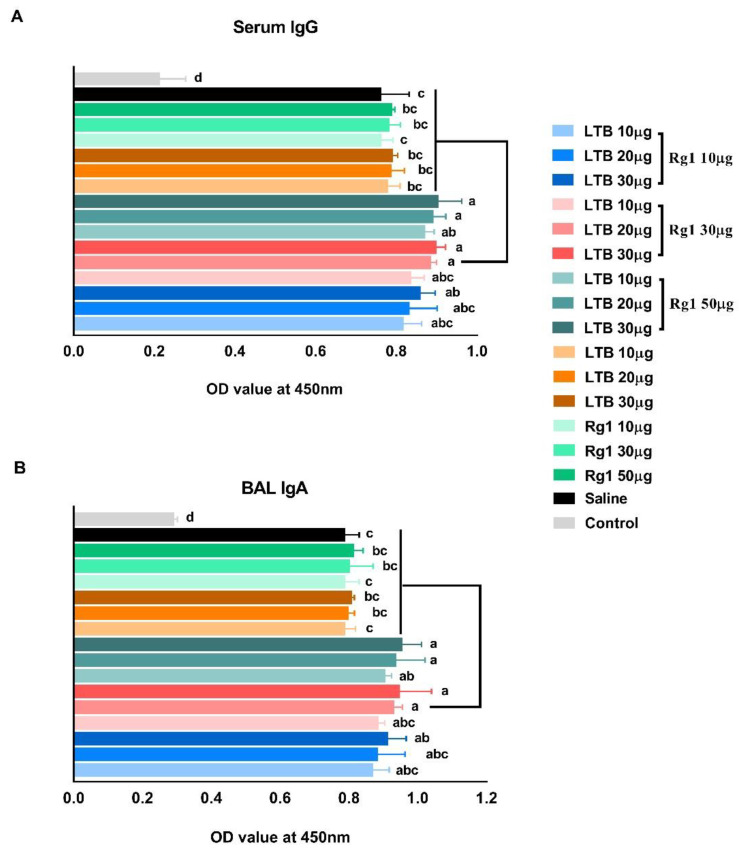
B subunit of *Escherichia coli* heat-labile enterotoxin (LTB-Rg1) increased porcine reproductive and respiratory syndrome virus (PRRSV)-specific antibody responses. After anesthesia with isoflurane, mice (*n* = 6/group) were intranasally immunized with an inactivated PRRSV vaccine (1 × 10^5^ TCID_50_) admixed with saline, LTB alone (10–30 µg), Rg1 alone (10–50 µg), or LTB (10–30 µg) combined with Rg1 (10–50 µg) on days 0, 7, and 21. Non-immunized mice served as negative controls. (**A**) Sera were collected on day 28 for IgG antibody analysis. (**B**) BAL fluid was collected on day 28 for IgA antibody analysis. Data are expressed as means ± SD. Different letters represent significant difference (*p* < 0.05).

To evaluate the synergistic adjuvant effect of LTB and Rg1 on inactivated PRRSV vaccine, mice were intranasally immunized as described in experiment A. PRRSV-specific IgG antibody responses in serum (Figure 2A) and IgA antibody responses in BAL fluid (Figure 2B) were analyzed 28 days after primary immunization. The addition of LTB or Rg1 alone did not significantly enhance PRRSV-specific antibody responses, while the addition of LTB and Rg1 together enhanced both serum IgG and respiratory IgA responses at various combination concentrations tested in a dose-dependent manner. Specifically, mice receiving vaccine adjuvanted with 20 or 30 µg of LTB plus 30 or 50 µg of Rg1 generated significantly higher PRRSV-specific IgG and IgA antibody responses compared to vaccine alone or vaccine admixed with one adjuvant. In view of efficacy, safety, and economic cost, 20 µg of LTB plus 30 µg of Rg1 (LTB-Rg1) was chosen for the optimal combination. 

### 3.2. LTB-Rg1 Induces Earlier and Prolonged Antibody Responses 

**Figure 3:**
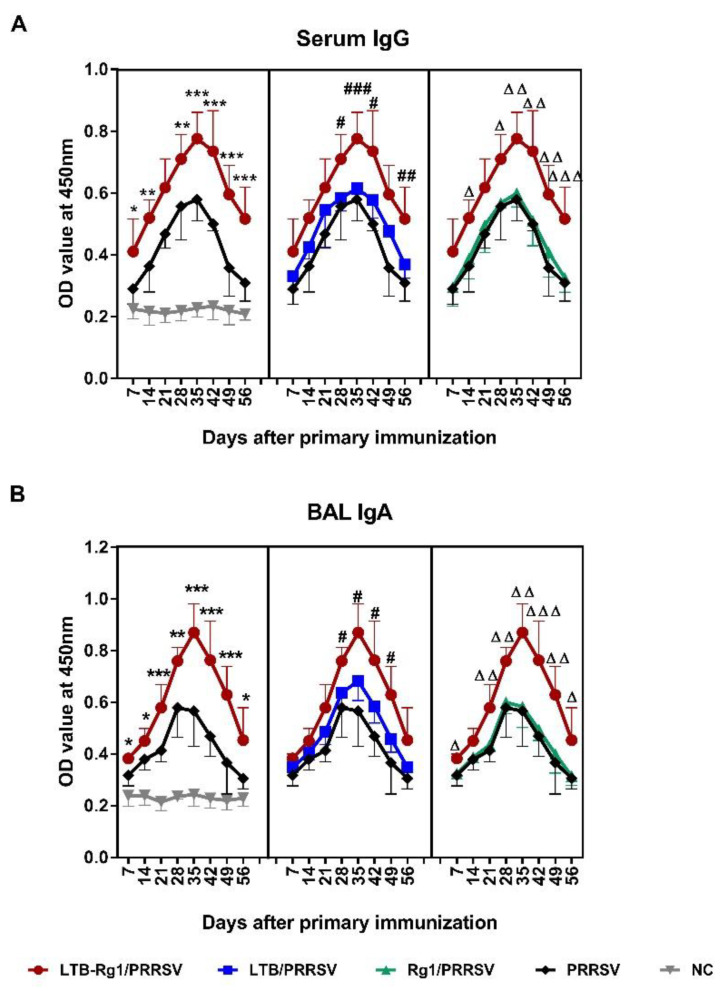
LTB-Rg1 significantly increased PRRSV-specific serum IgG and respiratory IgA responses. Mice (*n* = 48/group) were immunized intranasally with PRRSV vaccine admixed with saline, LTB, Rg1, or LTB-Rg1. Non-immunized mice served as negative controls. On days 7, 14, 21, 28, 35, 42, 49, and 56, 6 mice were taken out from each group and euthanized. (**A**) Sera were collected to detect IgG response. (**B**) BAL fluid was collected to detect IgA response. Sub-plots show the comparison of LTB-Rg1/PRRSV with PRRSV, LTB/PRRSV, Rg1/PRRSV. Data are expressed as means ± SD. * *p* < 0.05, ** *p* < 0.01, *** *p* < 0.001 versus PRRSV group. ^#^ *p* < 0.05, ^##^ *p* < 0.01, ^###^ *p* < 0.001 versus LTB/PRRSV group. ^∆^ *p* < 0.05, ^∆∆^ *p* < 0.01, ^∆∆∆^ *p* < 0.001 versus Rg1/PRRSV group.

**Figure 4:**
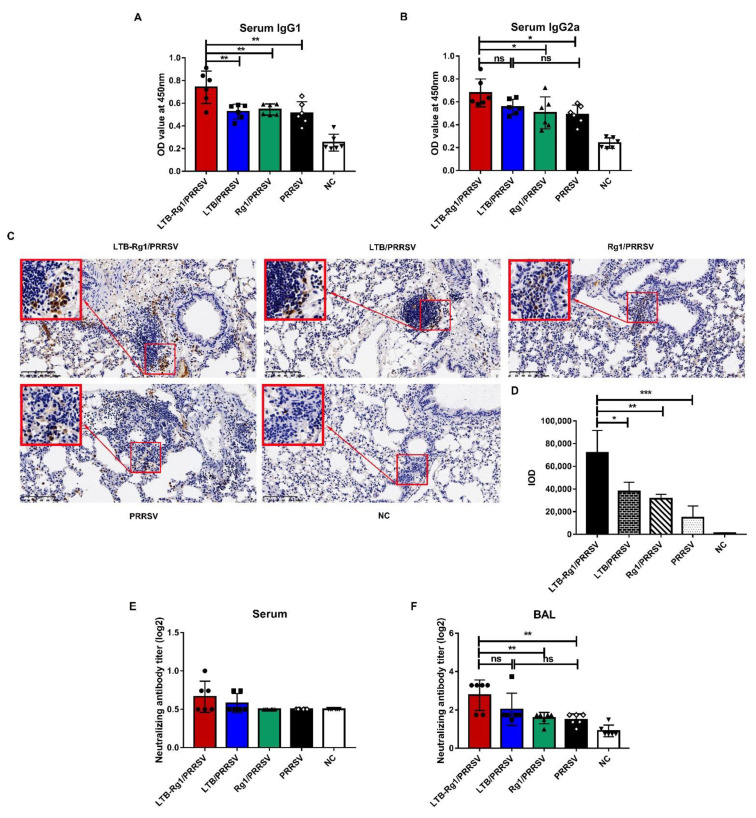
LTB-Rg1 significantly increased IgG isotypes, IgA-secreting plasma cells, and neutralizing antibody titers. Mice (*n* = 6/group) were immunized intranasally with PRRSV vaccine admixed with saline, LTB, Rg1, or LTB-Rg1 and euthanized on day 35 post-priming. Non-immunized mice served as negative controls. (**A**,**B**) Sera were collected for determination of IgG1 and IgG2a responses. (**C**) Lung tissues were harvested for detection of IgA-secreting cells by immunohistochemical staining. (**D**) The integrated optical density (IOD) was analyzed. (**E**,**F**) Serum samples and BAL fluid were tested for neutralizing antibody titer. Data are expressed as means ± SD. * *p* < 0.05, ** *p* < 0.01, *** *p* < 0.001. ns, no significant difference.

To further assess the effectiveness of LTB-Rg1 on the induction of PRRSV-specific antibody responses, serum and BAL fluid from mice were analyzed at indicated time points after primary immunization (experiment B). Immunization of mice with PRRSV vaccine supplemented with LTB-Rg1 (LTB-Rg1/PRRSV) over a 56-day immunization course induced stronger serum IgG and respiratory IgA antibody responses than immunization with vaccine alone or vaccine admixed with individual adjuvant (Figure 3A,B). Notably, the addition of LTB-Rg1 increased antibody responses as early as 7 days post-priming; the highest responses were achieved on day 35, with prominent serum IgG1 and IgG2a isotypes (Figure 4A,B) and adequate levels of pulmonary IgA-secreting cells (Figure 4C,D). These elevated immune responses lasted longer than the 56-day experimental period. However, little enhancement was found in mice treated with vaccine plus only one adjuvant. 

### 3.3. LTB-Rg1 Increases Neutralizing Antibody Titers 

To investigate neutralizing antibody titer, serum and BAL fluid were collected 35 days after primary immunization (experiment C). As shown in Figure 4E,F, immunization with LTB-Rg1/PRRSV elicited a stronger BAL neutralizing antibody response than that of either the vaccine-only or Rg1/PRRSV group. Notably, no significant difference was observed between LTB-Rg1/PRRSV and LTB/PRRSV treatment, although LTB/PRRSV had no significant effect compared to the vaccine-only group. Moreover, none of the treatments showed a significant enhancement in serum-neutralizing antibody response compared to the vaccine-only group. 

### 3.4. LTB-Rg1 Induces Higher Lymphocyte Proliferation

**Figure 5:**
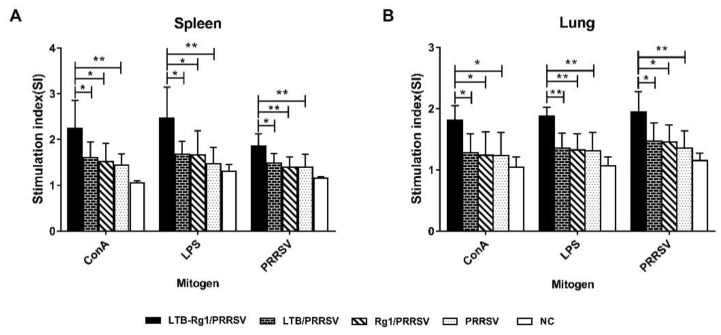
LTB-Rg1 significantly enhanced lymphocyte proliferative response to ConA, LPS, and PRRSV antigen. Mice (*n* = 6/group) were immunized intranasally with PRRSV vaccine combined with saline, LTB, Rg1, or LTB-Rg1. Non-immunized mice served as negative controls. Splenocytes and lung mononuclear cells were collected on day 35 and stimulated with ConA, LPS, or PRRSV antigen for 48h. (**A**) Splenocyte proliferation was determined using CCK-8. (**B**) The proliferation of lung mononuclear cells was measured. Stimulation index (SI) was calculated. Data are expressed as means ± SD. * *p* < 0.05, ** *p* < 0.01.

The proliferation of T and B lymphocytes is an important indicator of cell-mediated immune response. To compare the ability of PRRSV vaccine with or without LTB-Rg1 to induce cell-mediated immune responses, mononuclear cells isolated from the spleen and lung tissues from the immunized mice were stimulated with ConA, LPS, and PRRSV antigen (experiment C). As shown in Figure 5A,B, mice immunized with LTB-Rg1/PRRSV exhibited higher lymphocyte proliferative responses to ConA, LPS, and PRRSV antigen stimulation than mice immunized either with vaccine alone or vaccine admixed with individual adjuvant. However, no significant potentiation was observed in either of the single-adjuvant groups, demonstrating the clear advantage of using the combined adjuvant to elicit a robust cell-mediated immune response. 

### 3.5. LTB-Rg1 Selectively Expanded CD4^+^ T Cell Proliferation

**Figure 6:**
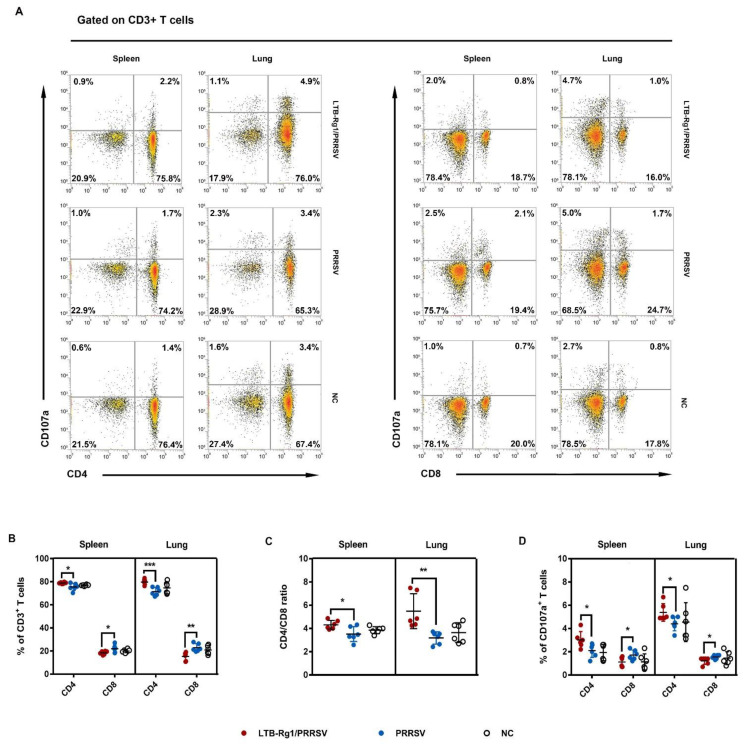
LTB-Rg1 selectively expanded CD4^+^ T cell proliferation. Mice (*n* = 6/group) received PRRSV vaccine without or with LTB-Rg1. Splenocytes and lung mononuclear cells were collected on day 35 post-priming and analyzed by flow cytometry. (**A**,**B**) CD4^+^ and CD8^+^ T cell subpopulations. (**C**) CD4^+^/CD8^+^ ratio. (**D**) CD107a^+^CD4^+^ and CD107a^+^CD8^+^ T cell percentages. Data are expressed as means ± SD. * *p* < 0.05, ** *p* < 0.01, *** *p* < 0.001.

T lymphocyte (CD3^+^) subpopulations in spleen and lung tissues were further evaluated after the sacrifice of mice on day 35 (experiment D). As shown in Figure 6A,B, the percentage of CD4^+^ cells in either splenic or pulmonary CD3^+^ T lymphocytes was significantly increased after administration of LTB-Rg1/PRRSV, compared to the vaccine-only group, while the frequency of CD8^+^ cells was decreased. The CD4^+^ /CD8^+^ ratio is an important indicator of the state of equilibrium of immune responses against antigen [28]. Higher CD4^+^ /CD8^+^ ratios were found in splenic and pulmonary T lymphocytes from mice immunized with LTB-Rg1/PRRSV when compared with those in mice receiving only the vaccine (Figure 6C). Because CD107a cell surface expression has been described as a marker of cytotoxic T cell activation [29], CD107a expression was analyzed in CD4^+^ and CD8^+^ T cells from immunized mice. As shown in Figure 6D, LTB-Rg1/PRRSV vaccination increased CD107a expression in CD4^+^ T cells from spleen and lung tissues, but decreased the percentage of CD107a^+^CD8^+^ T cells, compared to mice treated with the vaccine alone.

### 3.6. LTB-Rg1 Enhances Th1, Th2, and Th17 Cellular Immunity

**Figure 7:**
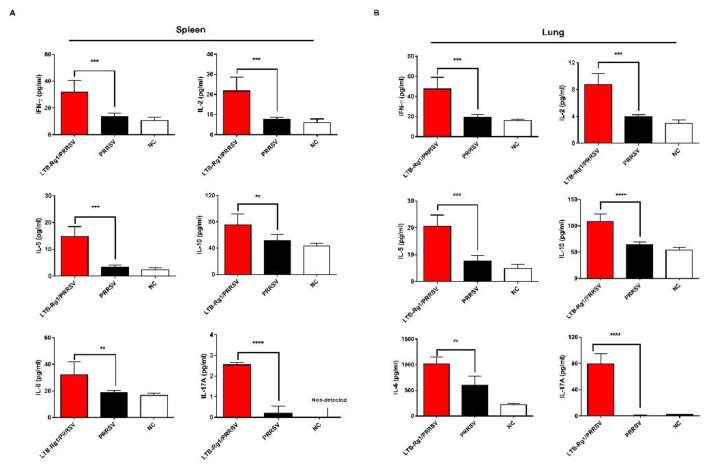
LTB-Rg1 significantly increased cytokine production. Mice (*n* = 6/group) received PRRSV vaccine without or with LTB-Rg1. Non-immunized animals served as negative controls. On day 35 post-priming, splenocytes and lung cells were prepared and co-cultured with PRRSV antigen for 48 h. (**A**) Cytokines in the supernatant of splenocytes were tested using ELISA. (**B**) Cytokines in the supernatant of lung cells were measured. Data are expressed as means ± SD. ** *p* < 0.01, *** *p* < 0.001, **** *p* < 0.0001.

**Figure 8:**
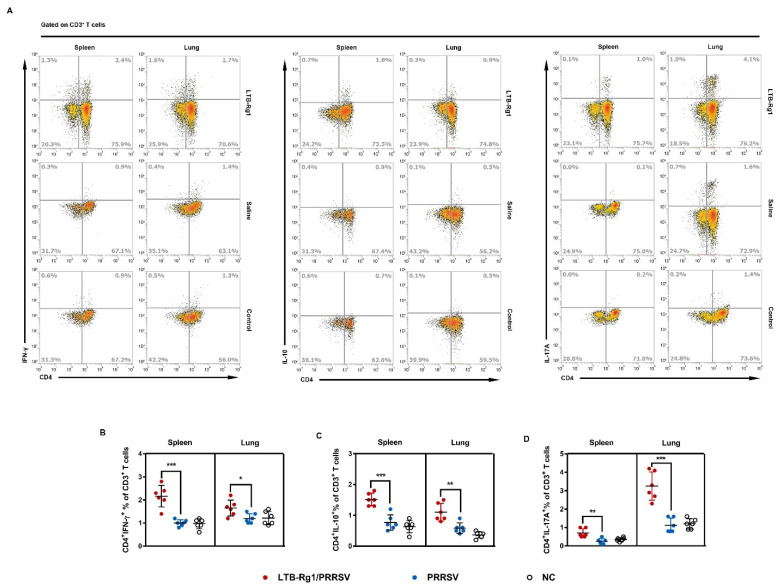
LTB-Rg1 significantly increased cytokine-producing T cells. Mice (*n* = 6/group) received PRRSV vaccine without or with LTB-Rg1on days 0, 7, and 21. On day 35, splenocytes and lung mononuclear cells were collected and analyzed by flow cytometry. (**A**,**B**) Percentages of interferon (IFN)-γ^+^CD4^+^ T cells. (**A**,**C**) Percentages of IL-10^+^CD4^+^ T cells. (**A**,**D**) Percentages of IL-17A^+^CD4^+^ T cells. Data are expressed as means ± SD. * *p* < 0.05, ** *p* < 0.01, *** *p* < 0.001.

Antigen-specific CD4^+^ T cells can be divided into multiple subsets producing various cytokines following antigen stimulation. We further evaluated mononuclear cells isolated from spleen and lung tissues from immunized mice for cytokine production upon PRRSV antigen re-stimulation in vitro. Our data demonstrate that cells from mice immunized with LTB-Rg1/PRRSV produce higher levels of Th1 (IFN-γ and IL-2), Th2 (IL-5 and IL-10), and Th17 (IL-6 and IL-17A) cytokines than cells from mice immunized with the vaccine alone (Figure 7A,B). To detect cell subsets responding to PRRSV antigen, we examined lineage differentiation of cytokine-producing cells. The frequencies of IFN-γ-, IL-10-, and IL-17A-producing CD4^+^ T cells in spleen and lung tissues from mice immunized with LTB-Rg1/PRRSV were significantly higher than those induced by vaccine-only treatment (Figure 8A–D), a finding that is consistent with the presence of cytokine production. 

### 3.7. LTB-Rg1 Up-Regulates Type I Interferon Signaling Pathway

**Figure 9:**
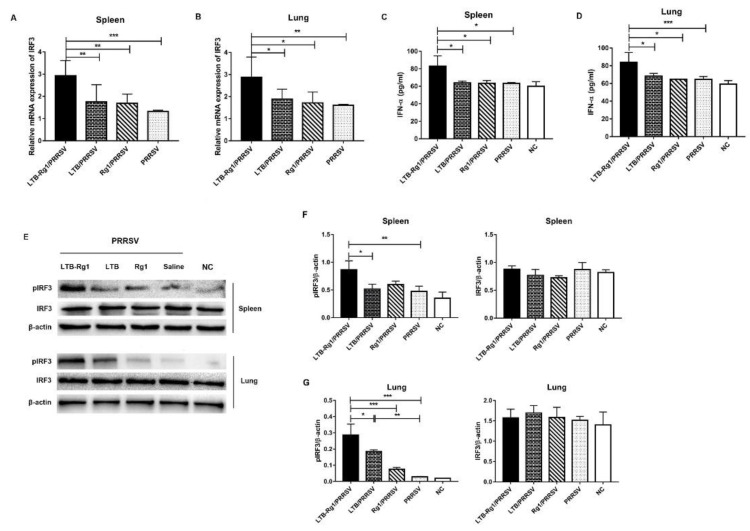
LTB-Rg1 up-regulated type I interferon signaling pathway. Mice (*n* = 6/group) were immunized intranasally with PRRSV vaccine admixed with either saline, LTB, Rg1, or LTB-Rg1. On day 35, splenocytes and lung mononuclear cells were collected. (**A**,**B**) One aliquot each of splenocytes and lung mononuclear cells was re-stimulated with PRRSV antigen for 24 h and tested for interferon regulatory factor (IRF) 3 mRNA expression by qPCR. (**C**,**D**) The remaining spleen and lung cells were re-stimulated with PRRSV antigen for 72 h and tested for IFN-α quantification in the supernatant by ELISA. (**E**) Cell lysis was analyzed for phosphorylation of IRF3 by Western blot. (**F**,**G**) The intensity ratios of pIRF3 and IRF3 bands normalized by β-actin in spleen and lung tissues. Data are expressed as means ± SD. * *p* < 0.05, ** *p* < 0.01, *** *p* < 0.001.

The immediate innate immune response to invading virus is the rapid induction of type I interferon, which is crucial for triggering an effective antiviral response and immunity [30]. To examine the effect of LTB-Rg1 on type I interferon signaling activation, mononuclear cells from spleen and lung tissues of immunized mice were harvested on day 35 after primary immunization and re-stimulated with PRRSV antigen in vitro. The expression of interferon (IFN)-α and interferon regulatory Factor (IRF)-3 were analyzed. As shown in Figure 9 A–D, the addition of LTB-Rg1 significantly up-regulated mRNA expression of IRF-3 and increased the secretion of IFN-α from splenocytes and lung cells upon antigen re-stimulation, compared to vaccine-only or single-adjuvant treatment. This finding was further confirmed by Western blot analysis that showed stronger bands of phosphorylated IRF3 in splenocytes and lung cells from mice immunized with LTB-Rg1/PRRSV compared to those from mice receiving vaccine alone or vaccine admixed with a single adjuvant (Figure 9E–G and supplementary Figure S1). 

## 4. Discussion

To date, vaccination to prevent PRRSV infection has achieved little success, as the current vaccines fail to induce sufficient immunity and have limited cross-protective efficacy. One strategy to solve this problem potentially involves the use of adjuvant in vaccine formulations to improve vaccination efficacy. Mucosal immunity contributes a vital role in immune defenses against PRRSV infection. However, parenteral vaccination is generally unable to induce sufficient mucosal immunity. In the present study, we combined two classes of adjuvants, recombinant LTB and ginsenoside Rg1, and sought to employ a combined adjuvant approach to develop an improved mucosal adjuvant. We found that this combined adjuvant, when given with an inactivated PRRSV vaccine intranasally, promoted multifaceted PRRSV-specific systemic and mucosal immune responses, including IgG and IgA antibodies, neutralizing antibodies, lymphocyte proliferation and Th1-, Th2-, Th17-type CD4^+^ T cell activities.

Even though LTB has been used widely as an adjuvant [19,31,32], IN administration of LTB has not overcome the major limitation of transient adverse effects (Bell’s palsy) in humans [33]. Earlier studies in animal models did not indicate any adverse effects in laboratory animals [11] and demonstrated that IN administration of nontoxic LT was safe without any pathological changes in the nasal mucosa of mice 3 days after administration [34,35]. Intracerebral injections of LTB at doses lower than 22.7 µg/mouse did not cause any significant adverse effects in brain tissues in mice [35]. The present study showed that LTB treatment at safe doses (lower than 30 µg/mouse) administered via the IN route failed to induce any enhancement in either local or systemic antibody responses to the PRRSV vaccine. Interestingly, those LTB doses did show synergistic effects when administered as an adjuvant together with Rg1 in mice, showing earlier, stronger, and more prolonged duration of both serum IgG and respiratory IgA antibody responses compared to those elicited by vaccine-only immunization. Moreover, the use of the combined LTB-Rg1 adjuvant also showed great advantages over treatment with either adjuvant alone. Mucosal IgA, one of the most critical immunoglobulins, acts as the first defense against pathogen infection [36]. The combined adjuvant significantly increased IgA-secreting plasma cells in the lung tissues on day 35 post-priming, a result consistent with the finding of higher levels of IgA and neutralizing antibody in BAL fluid compared with the vaccine-only group. Although neutralizing antibody generated in the respiratory tract is very valuable since that location is the first line of defense against invasion of deeper tissues by the virus, it is important to note that a specific titer of neutralizing antibody is required to prevent PRRSV infection. Early experiments on prophylactic administration of neutralizing antibody in animals indicated that a neutralizing antibody titer greater than 3 (log_2_) can serve as a correlate of protection [37,38]. However, in our study, this combined adjuvant could not give rise to protective levels of neutralizing antibodies in either serum or respiratory tract. Interestingly, even though neutralizing antibodies have received the most attention in PRRSV immunology, pigs are known to control infections in the absence of neutralizing antibodies [39,40]. Therefore, there must be other facets of the immune system which effectively control infection, such as non-neutralizing antibodies and T cell responses. 

Lymphocytes play a critical role in many aspects of antiviral immunity, including the activation of antibody production, determination of the cytokine milieu in the microenvironment of antigen presentation, killing of virally infected cells, and regulation of immune responses [41,42]. Compared to mice immunized with vaccine alone, improved proliferative responses to ConA, LPS, and PRRSV antigen were observed in splenic and pulmonary lymphocytes from mice immunized with LTB-Rg1/PRRSV, indicating that both T and B lymphocytes were activated by this treatment. Clonal expansion is essential for triggering B lymphocytes to produce antibodies. The enhanced lymphocyte proliferation to PRRSV antigen was consistent with the elevated PRRSV-specific IgG and IgA responses seen in mice immunized with LTB-Rg1/PRRSV. The predominant IgG subclass changes progressively in the immune response, which is dependent on the pattern of cytokines secreted by CD4^+^ T cells [43]. In mice, IL-5 and IL-10 preferentially convert activated B cells into the IgG1 isotype (Th2-type); IFN-γ and IL-2 selectively help the class switch to the IgG2a isotype (Th1-type) [44,45]. As reported in earlier research, PRRSV-infected pigs typically show insufficient Th1-type immune responses, leading to prolonged immunosuppression [46]. Administration of 50 µg/mouse dose of LTB enhanced the PRRSV-specific immune response skewed toward Th1 production, as previously observed [19]. Notably, in the current study, LTB combined with Rg1 significantly enhanced both IgG1 and IgG2a responses, a finding that may be correlated with the increased numbers of IFN-γ- and IL-10- producing CD4^+^ T cells and up-regulated IFN-γ, IL-2, IL-5, and IL-10 responses in the spleen and lung tissues. These results suggest that the combined adjuvant stimulates an unbiased Th1 and Th2 immune response. The Th17 response, a newly appreciated arm of the adaptive immune response, plays an important role in promoting effective immunity and combating secondary bacterial infections associated with PRRSV, particularly in mucosal tissue [47,48]. As previously reported, Th17 cytokines function in promoting mucosal antibody responses by up-regulating polymeric Ig receptor levels in epithelia, increasing transport of secretory IgA into the lumen of mucosal tissues, and enhancing B-cell differentiation into IgA-secreting cells in a T cell-independent way [49,50,51]. As observed, IN immunization of LTB-Rg1/PRRSV was effective in stimulating CD4^+^ T cells that produce Th17-related cytokines, such as IL-6 and IL-17, in parallel with the enhanced mucosal immune responses described above. 

The ratio of CD4^+^/CD8^+^ is a boundary marker of the status of the immune system, and higher CD4^+^/CD8^+^ ratios indicate a stronger immune response [52,53]. Previous research identified an early, large, and transient decrease in the CD4^+^/CD8^+^ T cell ratio during the course of PRRSV infection, which could be due either to a temporary loss of CD4^+^ T cells through apoptosis or to an increase in CD8^+^ T cells by antigen-specific proliferation [54]. Our data showed that the percentage of CD4^+^ T cells and ratio of CD4^+^/CD8^+^ were significantly increased in splenocytes and lung cells with the use of LTB-Rg1/PRRSV compared to the vaccine-only group. However, the proportion of CD8^+^ T cells was also decreased by the combined adjuvant, perhaps because of the effect of LTB on depletion of CD8^+^ T cells, as previously demonstrated [55]. It is important to note that the vaccine-induced CD4^+^ T cells could be preferential targets for immunosuppressive viruses such as the AIDS virus [56]. It has been reported that vaccine-elicited CD107a^-^CD4^+^ T cells are largely depleted in the acute phase of HIV infection, while the virus-specific CD107a^+^ CD4^+^ T cells, associated with cytotoxic CD4^+^ T-cell function via cytotoxic granules, are relatively resistant to depletion after challenge [56,57]. More importantly, cytotoxic CD4^+^ T cells have been found to be associated with control of early HIV replication and delay in disease progression [57,58,59], implying that induction of these cells may be an alternative approach for the control of immunosuppressive viruses, including PRRSV. In the present study, CD107a expression was strikingly enhanced in CD4^+^ T cells from mice immunized with LTB-Rg1/PRRSV when compared with the vaccine-only group; this effect may contribute to the lysis of infected antigen-presenting cells after PRRSV infection. The low population of CD107a^+^CD8^+^ T cells in the treatment of LTB-Rg1/PRRSV may be due to the loss of CD8^+^ T cells caused by LTB. Although the mechanism of PRRSV to escape host antiviral immune responses has not yet been well characterized, several in vivo experiments have pointed out that PRRSV possesses inhibitory effects on type I interferon responses during the early stages of infection; this inhibition is caused by multiple PRRSV proteins such as nonstructural protein (nsp) 1, nsp 2, nsp 4, nsp 5, nsp 11 and nucleocapsid [60,61,62,63,64,65]. Furthermore, the suppression of type I interferons in swine after exposure to PRRSV provides a favorable environment for virus replication and survival in phagocytic cells [54]. Since IFN-α up-regulates IFN-γ gene expression, a deficiency in IFN-α production will ultimately impair adaptive immunity, specifically, T cell-mediated IFN-γ responses and antiviral immune defenses [42]. The present study demonstrates that LTB-Rg1 enhances activity of IRF3, an important transcriptional factor in IFN-α induction, at both transcriptional and post-transcriptional levels, subsequently leading to elevated secretion of IFN-α. 

## 5. Conclusions

In conclusion, 20 µg of LTB combined with 10 µg of ginsenoside Rg1 as an intranasal adjuvant of PRRSV inactivated vaccine offers a promising strategy for improved protection against PRRSV in mice. The combined use of these adjuvants with the PRRSV vaccine activated the type I interferon signaling pathway and induced significantly stronger systemic and mucosal immune responses than the administration of the vaccine alone. These findings demonstrate the advantages of combined adjuvants over the use of single adjuvants in boosting the effectiveness of PRRSV vaccines. Further study is required to evaluate the efficacy of this combination adjuvant on PRRSV vaccination in swine and to explore the detailed molecular mechanisms underlying the combined-adjuvant effects that we have observed. 

## Data Availability

The data is available from the corresponding author upon request.

## Supplementary Materials

The following are available online at https://www.mdpi.com/2076-393X/9/3/266/s1.

## References

[1] Evans A.B., Loyd H., Dunkelberger J.R., van Tol S., Bolton M.J., Dorman K.S., Dekkers J.C.M., Carpenter S. (2017). Antigenic and Biological Characterization of ORF2-6 Variants at Early Times Following PRRSV Infection. Viruses.

[2] Nieuwenhuis N., Duinhof T.F., van Nes A. (2012). Economic analysis of outbreaks of porcine reproductive and respiratory syndrome virus in nine sow herds. Vet. Rec..

[3] Wang H., Du L., Liu F., Wei Z., Gao L., Feng W.H. (2019). Highly Pathogenic Porcine Reproductive and Respiratory Syndrome Virus Induces Interleukin-17 Production via Activation of the IRAK1-PI3K-p38MAPK-C/EBPbeta/CREB Pathways. J. Virol..

[4] Lunney J.K., Fang Y., Ladinig A., Chen N., Li Y., Rowland B., Renukaradhya G.J. (2016). Porcine Reproductive and Respiratory Syndrome Virus (PRRSV): Pathogenesis and Interaction with the Immune System. Annu. Rev. Anim. Biosci..

[5] Linhares D.C., Cano J.P., Wetzell T., Nerem J., Torremorell M., Dee S.A. (2012). Effect of modified-live porcine reproductive and respiratory syndrome virus (PRRSv) vaccine on the shedding of wild-type virus from an infected population of growing pigs. Vaccine.

[6] Renukaradhya G.J., Meng X.J., Calvert J.G., Roof M., Lager K.M. (2015). Live porcine reproductive and respiratory syndrome virus vaccines: Current status and future direction. Vaccine.

[7] Renukaradhya G.J., Meng X.J., Calvert J.G., Roof M., Lager K.M. (2015). Inactivated and subunit vaccines against porcine reproductive and respiratory syndrome: Current status and future direction. Vaccine.

[8] Binjawadagi B., Dwivedi V., Manickam C., Ouyang K., Wu Y., Lee L.J., Torrelles J.B., Renukaradhya G.J. (2014). Adjuvanted poly(lactic-co-glycolic) acid nanoparticle-entrapped inactivated porcine reproductive and respiratory syndrome virus vaccine elicits cross-protective immune response in pigs. Int. J. Nanomed..

[9] Alshweiat A., Ambrus R., Csoka I. (2019). Intranasal Nanoparticulate Systems as Alternative Route of Drug Delivery. Curr. Med. Chem..

[10] Lei H., Peng X., Shu H., Zhao D. (2015). Intranasal immunization with live recombinant Lactococcus lactis combined with heat-labile toxin B subunit protects chickens from highly pathogenic avian influenza H5N1 virus. J. Med. Virol..

[11] Ma Y. (2016). Recent advances in nontoxic Escherichia coli heat-labile toxin and its derivative adjuvants. Expert Rev. Vaccines.

[12] Newsted D., Fallahi F., Golshani A., Azizi A. (2015). Advances and challenges in mucosal adjuvant technology. Vaccine.

[13] Liu L., Ma Y., Zhou H., Wu M. (2016). Quantitative Proteomic Analysis of Escherichia coli Heat-Labile Toxin B Subunit (LTB) with Enterovirus 71 (EV71) Subunit VP1. Int. J. Mol. Sci..

[14] Marchioro S.B., Fisch A., Gomes C.K., Jorge S., Galli V., Haesebrouck F., Maes D., Dellagostin O., Conceicao F.R. (2014). Local and systemic immune responses induced by a recombinant chimeric protein containing Mycoplasma hyopneumoniae antigens fused to the B subunit of Escherichia coli heat-labile enterotoxin LTB. Vet. Microbiol..

[15] Thanasarasakulpong A., Poolperm P., Tankaew P., Sawada T., Sthitmatee N. (2015). Protectivity conferred by immunization with intranasal recombinant outer membrane protein H from Pasteurella multocida serovar A:1 in chickens. J. Vet. Med. Sci..

[16] Duan Q., Xia P., Nandre R., Zhang W., Zhu G. (2019). Review of Newly Identified Functions Associated With the Heat-Labile Toxin of Enterotoxigenic Escherichia coli. Front. Cell Infect. Microbiol..

[17] Aman A.T., Fraser S., Merritt E.A., Rodigherio C., Kenny M., Ahn M., Hol W.G., Williams N.A., Lencer W.I., Hirst T.R. (2001). A mutant cholera toxin B subunit that binds GM1- ganglioside but lacks immunomodulatory or toxic activity. Proc. Natl. Acad. Sci. USA.

[18] Zoeteweij J.P., Epperson D.E., Porter J.D., Zhang C.X., Frolova O.Y., Constantinides A.P., Fuhrmann S.R., El-Amine M., Tian J.H., Ellingsworth L.R. (2006). GM1 binding-deficient exotoxin is a potent noninflammatory broad spectrum intradermal immunoadjuvant. J. Immunol..

[19] Su F., Xu L., Xue Y., Li J., Fu Y., Yu B., Wang S., Yuan X. (2019). Th1-biased immunoadjuvant effect of the recombinant B subunit of an Escherichia coli heat-labile enterotoxin on an inactivated porcine reproductive and respiratory syndrome virus antigen via intranasal immunization in mice. J. Vet. Med. Sci..

[20] Cheng Y., Shen L.H., Zhang J.T. (2005). Anti-amnestic and anti-aging effects of ginsenoside Rg1 and Rb1 and its mechanism of action. Acta Pharmacol. Sin..

[21] Su F., Yuan L., Zhang L., Hu S. (2012). Ginsenosides Rg1 and Re act as adjuvant via TLR4 signaling pathway. Vaccine.

[22] Su F., Xue Y., Wang Y., Zhang L., Chen W., Hu S. (2015). Protective effect of ginsenosides Rg1 and Re on lipopolysaccharide-induced sepsis by competitive binding to Toll-like receptor 4. Antimicrob. Agents Chemother..

[23] Li X., Zhang Y.K., Yin B., Liang J.B., Jiang F., Wu W.X. (2019). Toll-Like Receptor 2 (TLR2) and TLR4 Mediate the IgA Immune Response Induced by Mycoplasma hyopneumoniae. Infect. Immun..

[24] Pandey M.K., Sung B., Ahn K.S., Kunnumakkara A.B., Chaturvedi M.M., Aggarwal B.B. (2007). Gambogic acid, a novel ligand for transferrin receptor, potentiates TNF-induced apoptosis through modulation of the nuclear factor-kappaB signaling pathway. Blood.

[25] Gao X., Zhao L., Wang S., Yang J., Yang X. (2013). Enhanced inducible costimulator ligand (ICOS-L) expression on dendritic cells in interleukin-10 deficiency and its impact on T-cell subsets in respiratory tract infection. Mol. Med..

[26] Wang Y., Cui X., Yuan L., Maqbool B., Xu W., He S., Guan R., Hu S. (2020). A Solution with Ginseng Saponins and Selenium as Vaccine Diluent to Increase Th1/Th2 Immune Responses in Mice. J. Immunol. Res..

[27] Livak K.J., Schmittgen T.D. (2001). Analysis of relative gene expression data using real-time quantitative PCR and the 2(-Delta Delta C(T)) Method. Methods.

[28] Sainz T., Serrano-Villar S., Diaz L., Gonzalez Tome M.I., Gurbindo M.D., de Jose M.I., Mellado M.J., Ramos J.T., Zamora J., Moreno S. (2013). The CD4/CD8 ratio as a marker T-cell activation, senescence and activation/exhaustion in treated HIV-infected children and young adults. AIDS.

[29] Geffner L., Basile J.I., Yokobori N., Sabio Y.G.C., Musella R., Castagnino J., Sasiain M.C., de la Barrera S. (2014). CD4(+) CD25(high) forkhead box protein 3(+) regulatory T lymphocytes suppress interferon-gamma and CD107 expression in CD4(+) and CD8(+) T cells from tuberculous pleural effusions. Clin. Exp. Immunol..

[30] Wong P.T., Goff P.H., Sun R.J., Ruge M.J., Ermler M.E., Sebring A., O'Konek J.J., Landers J.J., Janczak K.W., Sun W. (2020). Combined Intranasal Nanoemulsion and RIG-I Activating RNA Adjuvants Enhance Mucosal, Humoral, and Cellular Immunity to Influenza Virus. Mol. Pharm..

[31] Ji J., Griffiths K.L., Milburn P.J., Hirst T.R., O’Neill H.C. (2015). The B subunit of *Escherichia coli* heat-labile toxin alters the development and antigen-presenting capacity of dendritic cells. J. Cell Mol. Med..

[32] Albert M.J., Haridas S., Ebenezer M., Raghupathy R., Khan I. (2015). Immunization with a Double-Mutant (R192G/L211A) of the Heat-Labile Enterotoxin of *Escherichia coli* Offers Partial Protection against Campylobacter jejuni in an Adult Mouse Intestinal Colonization Model. PLoS ONE.

[33] Lewis D.J., Huo Z., Barnett S., Kromann I., Giemza R., Galiza E., Woodrow M., Thierry-Carstensen B., Andersen P., Novicki D. (2009). Transient facial nerve paralysis (Bell’s palsy) following intranasal delivery of a genetically detoxified mutant of Escherichia coli heat labile toxin. PLoS ONE.

[34] Hagiwar Y., Tsuji T., Iwasaki T., Kadowaki S., Asanuma H., Chen Z., Komase K., Suzuki Y., Aizawa C., Kurata T. (2001). Effectiveness and safety of mutant Escherichia coli heat-labile enterotoxin (LT H44A) as an adjuvant for nasal influenza vaccine. Vaccine.

[35] Hagiwara Y., Iwasaki T., Asanuma H., Sato Y., Sata T., Aizawa C., Kurata T., Tamura S. (2001). Effects of intranasal administration of cholera toxin (or Escherichia coli heat-labile enterotoxin) B subunits supplemented with a trace amount of the holotoxin on the brain. Vaccine.

[36] Muir W.I., Bryden W.L., Husband A.J. (1998). Evaluation of the efficacy of intraperitoneal immunization in reducing Salmonella typhimurium infection in chickens. Poult. Sci..

[37] Li X., Galliher-Beckley A., Pappan L., Trible B., Kerrigan M., Beck A., Hesse R., Blecha F., Nietfeld J.C., Rowland R.R. (2014). Comparison of host immune responses to homologous and heterologous type II porcine reproductive and respiratory syndrome virus (PRRSV) challenge in vaccinated and unvaccinated pigs. Biomed. Res. Int..

[38] Plotkin S.A., Gilbert P.B. (2012). Nomenclature for immune correlates of protection after vaccination. Clin. Infect. Dis..

[39] Fontanella E., Ma Z., Zhang Y., de Castro A.M., Shen H., Halbur P.G., Opriessnig T. (2017). An interferon inducing porcine reproductive and respiratory syndrome virus vaccine candidate elicits protection against challenge with the heterologous virulent type 2 strain VR-2385 in pigs. Vaccine.

[40] Batista L., Pijoan C., Dee S., Olin M., Molitor T., Joo H.S., Xiao Z., Murtaugh M. (2004). Virological and immunological responses to porcine reproductive and respiratory syndrome virus in a large population of gilts. Can. J. Vet. Res..

[41] Barranco I., Gomez-Laguna J., Rodriguez-Gomez I.M., Quereda J.J., Salguero F.J., Pallares F.J., Carrasco L. (2012). Immunohistochemical expression of IL-12, IL-10, IFN-alpha and IFN-gamma in lymphoid organs of porcine reproductive and respiratory syndrome virus-infected pigs. Vet. Immunol. Immunopathol..

[42] Loving C.L., Osorio F.A., Murtaugh M.P., Zuckermann F.A. (2015). Innate and adaptive immunity against Porcine Reproductive and Respiratory Syndrome Virus. Vet. Immunol. Immunopathol..

[43] Hussain R., Menz B., Dockrell H.M., Chiang T.J. (1995). Recognition of Mycobacterium leprae recombinant 18,000 MW epitopes by IgG subclasses in leprosy. Immunology.

[44] Tangye S.G., Ferguson A., Avery D.T., Ma C.S., Hodgkin P.D. (2002). Isotype switching by human B cells is division-associated and regulated by cytokines. J. Immunol..

[45] Ni J., Bi S., Xu W., Zhang C., Lu Y., Zhai L., Hu S. (2016). Improved immune response to an attenuated pseudorabies virus vaccine by ginseng stem-leaf saponins (GSLS) in combination with thimerosal (TS). Antiviral. Res..

[46] Shi K.C., Guo X., Ge X.N., Liu Q., Yang H.C. (2010). Cytokine mRNA expression profiles in peripheral blood mononuclear cells from piglets experimentally co-infected with porcine reproductive and respiratory syndrome virus and porcine circovirus type 2. Vet. Microbiol..

[47] Zhang L., Zhou L., Ge X., Guo X., Han J., Yang H. (2016). The Chinese highly pathogenic porcine reproductive and respiratory syndrome virus infection suppresses Th17 cells response in vivo. Vet. Microbiol..

[48] Jie Z., Yang J.Y., Gu M., Wang H., Xie X., Li Y., Liu T., Zhu L., Shi J., Zhang L. (2018). NIK signaling axis regulates dendritic cell function in intestinal immunity and homeostasis. Nat. Immunol..

[49] Katakam A.K., Brightbill H., Franci C., Kung C., Nunez V., Jones C., Peng I., Jeet S., Wu L.C., Mellman I. (2015). Dendritic cells require NIK for CD40-dependent cross-priming of CD8+ T cells. Proc. Natl. Acad. Sci. USA.

[50] Vedantam G., Viswanathan V.K. (2011). Unlocking the gates to inflammatory bowel disease: The role of Enterococcus faecalis gelatinase. Gastroenterology.

[51] Zhou Y., Chen H., He H., Du Y., Hu J., Li Y., Li Y., Zhou Y., Wang H., Chen Y. (2016). Increased Enterococcus faecalis infection is associated with clinically active Crohn disease. Medicine (Baltimore).

[52] Jiang L., Yu Z., Lin Y., Cui L., Yao S., Lv L., Liu J. (2018). Low-molecular-weight polysaccharides from Agaricus blazei Murrill modulate the Th1 response in cancer immunity. Oncol. Lett..

[53] Serrano-Villar S., Sainz T., Lee S.A., Hunt P.W., Sinclair E., Shacklett B.L., Ferre A.L., Hayes T.L., Somsouk M., Hsue P.Y. (2014). HIV-infected individuals with low CD4/CD8 ratio despite effective antiretroviral therapy exhibit altered T cell subsets, heightened CD8+ T cell activation, and increased risk of non-AIDS morbidity and mortality. PLoS Pathog..

[54] Rahe M.C., Murtaugh M.P. (2017). Mechanisms of Adaptive Immunity to Porcine Reproductive and Respiratory Syndrome Virus. Viruses.

[55] Nashar T.O., Webb H.M., Eaglestone S., Williams N.A., Hirst T.R. (1996). Potent immunogenicity of the B subunits of Escherichia coli heat-labile enterotoxin: Receptor binding is essential and induces differential modulation of lymphocyte subsets. Proc. Natl. Acad. Sci. USA.

[56] Terahara K., Ishii H., Nomura T., Takahashi N., Takeda A., Shiino T., Tsunetsugu-Yokota Y., Matano T. (2014). Vaccine-induced CD107a+ CD4+ T cells are resistant to depletion following AIDS virus infection. J. Virol..

[57] Soghoian D.Z., Jessen H., Flanders M., Sierra-Davidson K., Cutler S., Pertel T., Ranasinghe S., Lindqvist M., Davis I., Lane K. (2012). HIV-specific cytolytic CD4 T cell responses during acute HIV infection predict disease outcome. Sci. Transl. Med..

[58] Johnson S., Eller M., Teigler J.E., Maloveste S.M., Schultz B.T., Soghoian D.Z., Lu R., Oster A.F., Chenine A.L., Alter G. (2015). Cooperativity of HIV-Specific Cytolytic CD4 T Cells and CD8 T Cells in Control of HIV Viremia. J. Virol..

[59] Laforge M., Silvestre R., Rodrigues V., Garibal J., Campillo-Gimenez L., Mouhamad S., Monceaux V., Cumont M.C., Rabezanahary H., Pruvost A. (2018). The anti-caspase inhibitor Q-VD-OPH prevents AIDS disease progression in SIV-infected rhesus macaques. J. Clin. Investig..

[60] Yang L., Zhang Y.J. (2017). Antagonizing cytokine-mediated JAK-STAT signaling by porcine reproductive and respiratory syndrome virus. Vet. Microbiol..

[61] Yang L., Wang R., Ma Z., Xiao Y., Nan Y., Wang Y., Lin S., Zhang Y.J. (2017). Porcine Reproductive and Respiratory Syndrome Virus Antagonizes JAK/STAT3 Signaling via nsp5, Which Induces STAT3 Degradation. J. Virol..

[62] Huang C., Zhang Q., Guo X.K., Yu Z.B., Xu A.T., Tang J., Feng W.H. (2014). Porcine reproductive and respiratory syndrome virus nonstructural protein 4 antagonizes beta interferon expression by targeting the NF-kappaB essential modulator. J. Virol..

[63] Beura L.K., Sarkar S.N., Kwon B., Subramaniam S., Jones C., Pattnaik A.K., Osorio F.A. (2010). Porcine reproductive and respiratory syndrome virus nonstructural protein 1beta modulates host innate immune response by antagonizing IRF3 activation. J. Virol..

[64] Sun Z., Li Y., Ransburgh R., Snijder E.J., Fang Y. (2012). Nonstructural protein 2 of porcine reproductive and respiratory syndrome virus inhibits the antiviral function of interferon-stimulated gene 15. J. Virol..

[65] Sun Y., Ke H., Han M., Chen N., Fang W., Yoo D. (2016). Nonstructural Protein 11 of Porcine Reproductive and Respiratory Syndrome Virus Suppresses Both MAVS and RIG-I Expression as One of the Mechanisms to Antagonize Type I Interferon Production. PLoS ONE.

