# Supplementary material for: Escherichia coli Heat-Labile Enterotoxin B Subunit Combined with Ginsenoside Rg1 as an Intranasal Adjuvant Triggers Type I Interferon Signaling Pathway and Enhances Adaptive Immune Responses to an Inactivated PRRSV Vaccine in ICR Mice
Source: Vaccines (Basel). 2021 Mar 16;9(3):266. doi: 10.3390/vaccines9030266 (PMC8002527; doi:10.3390/vaccines9030266)
Supplement: Supplementary file 1 [file vaccines-09-00266-s001.pdf]

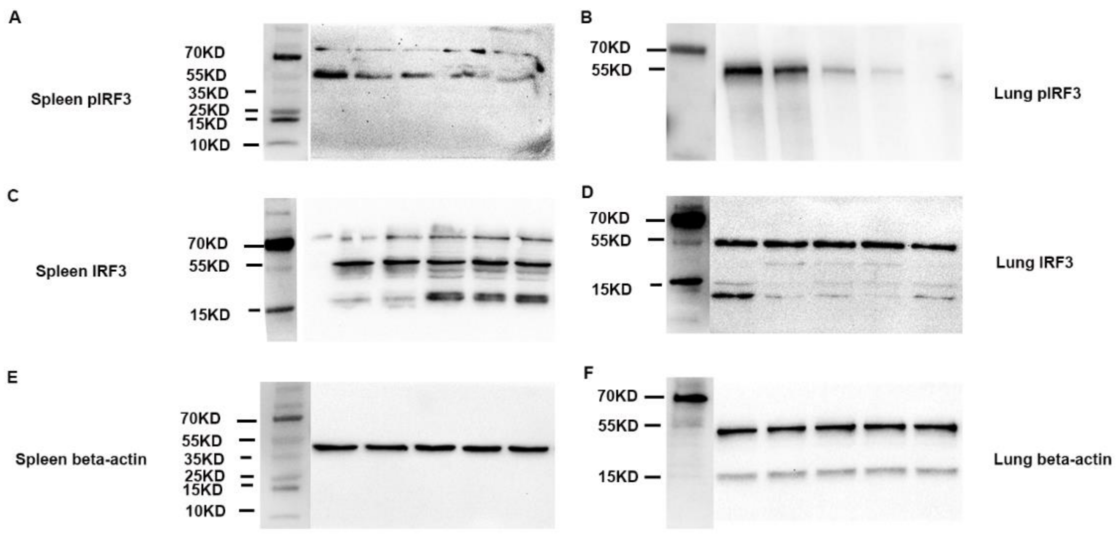

Figure S1. Original images of Western blot results shown in Figure 9E. (A) and (B) pIRF3 in spleen and lung tissues. (C) and (D) IRF3 in spleen and lung tissues. (E) and (F) β-actin in spleen and lung tissues.
